# New Metrics to Assess Type 2 Diabetes after Bariatric Surgery: The “Time-Within-Remission Range”

**DOI:** 10.3390/jcm9041070

**Published:** 2020-04-09

**Authors:** Ana de Hollanda, Albert Lecube, Miguel Angel Rubio, Enric Sánchez, Núria Vilarrasa, José Gregorio Oliva, María Luisa Fernández-Soto, Jordi Salas-Salvadó, María D. Ballesteros-Pomar, Andreea Ciudin, Ferran Torres, Concepción Vidal, María José Morales, Sergio Valdés, Silvia Pellitero, Inka Miñambres, Lluís Masmiquel, Albert Goday, Lorena Suarez, Liliam Flores, Marta Bueno, Assumpta Caixàs, Irene Bretón, Rosa Cámara, Romina Olbeyra, Rona Penso, María José de la Cruz, Andreu Simó-Servat, Francisca María Pereyra-García, Elena Teresa López-Mezquita, Anna Gils, Enzamaria Fidilio, Orosia Bandrés, Ángel Martínez, Jose Abuín, Montserrat Marques-Pamies, Laura Tuneu, Magdalena Arteaga, Olga Castañer, Fernando Goñi, Cristina Arrizabalaga, Manuel Antonio Botana, Alfonso Calañas, Ángel Rebollo

**Affiliations:** 1 Hospital Clínic de Barcelona, 08036 Barcelona, Spain; LFLORES@clinic.cat; 2 Institut d’Investigacions Biomèdiques August Pi i Sunyer (IDIBAPS), 08036 Barcelona, Spain; rominaolbeyra@gmail.com; 3 CIBER de Obesidad y Nutrición (CIBEROBN), Instituto de Salud Carlos III (ISCIII), 28029 Madrid, Spain; jordi.salas@urv.cat (J.S.-S.); Agoday@parcdesalutmar.cat (A.G.); 4 Hospital Universitari Arnau de Vilanova, Grup de Recerca en Obesitat, Diabetes i Metabolisme (ODIM), Institut de Recerca Biomèdica de Lleida (IRBLleida), Universitat de Lleida, 25198 Lleida, Spain; esanchez@irblleida.cat (E.S.); mbuenodiez@gmail.com (M.B.); 5 CIBER de Diabetes y Enfermedades Metabólicas Asociadas (CIBERDEM), Instituto de Salud Carlos III (ISCIII), 28029 Madrid, Spain; nuriavilarrasa@yahoo.es (N.V.); sergio.valdes@hotmail.es (S.V.); spelli75@gmail.com (S.P.); 6 Hospital Clínico San Carlos IDISSC, 28040 Madrid, Spain; marubioh@gmail.com; 7 Hospital Universitari de Bellvitge, Institut d’Investigació Biomèdica de Bellvitge (IDIBELL), L’Hospitalet de Llobregat, 08907 Barcelona, Spain; 8 Hospital Universitario Nuestra Señora de Candelaria, 38010 Santa Cruz de Tenerife, Spain; olivajg@gmail.com (J.G.O.); francispereyra@telefonica.net (F.M.P.-G.); 9 Hospital Universitario San Cecilio, 18016 Granada, Spain; marisafsoto@gmail.com (M.L.F.-S.); elopezmt@gmail.com (E.T.L.-M.); 10 Hospital Universitari Sant Joan de Reus, Institut d’Investigació Sanitària Pere Virgili (IISPV), Universitat Rovita i Virgili Reus, 43204 Tarragona, Spain; annagilsc@gmail.com; 11 Hospital de León, 24008 León, Spain; mdballesteros@telefonica.net; 12 Hospital Universitari Vall d’Hebron, 08035 Barcelona, Spain; aciudin@vhebron.net (A.C.); enzamariafidilio@gmail.com (E.F.); 13 Medical Statistics Core Facility, Clinical Pharmacology Deparment, Hospital Clínic Barcelona, 08036 Barcelona, Spain; Ferran.Torres@uab.cat; 14 Biostatistics Unit, Faculty of Medicine, Universitat Autònoma de Barcelona, 08193 Barcelona, Spain; 15 Hospital Royo Villanova, 50015 Zaragoza, Spain; cvidal@unizar.es (C.V.); orosiabandres@gmail.com (O.B.); 16 Complejo Hospitalario Universitario de Vigo, 36312 Vigo, Spain; maria.jose.morales.gorria@sergas.es (M.J.M.); angelmg88@hotmail.com (Á.M.); 17 Hospital Regional Universitario de Málaga, Instituto de Investigación Biomédica de Málaga (IBIMA), Universidad de Málaga, 29010 Málaga, Spain; jose.abuin.fdez@gmail.com; 18 Hospital Universitari Germans Trias i Pujol, Institut d’Investigació Germans Trias (IGTP), 08916 Badalona, Spain; montse98.89@gmail.com; 19 Hospital de la Santa Creu i Sant Pau, Universitat Autònoma de Barcelona, 08041 Barcelona, Spain; iminambres@santpau.cat (I.M.); laura.tuneu@quironsalud.es (L.T.); 20 Hospital Universitario Son Llàtzer, Instituto de Investigación Sanitaria de las Islas Baleares (IdISBa), 07198 Palma de Mallorca, Spain; lmasmiquel@gmail.com (L.M.); marteagao@gmail.com (M.A.); 21 Parc de Salut Mar, Institut Hospital del Mar d’Investigacions Mèdiques (IMIM), Universitat Autònoma de Barcelona, 08003 Barcelona, Spain; ocastaner@imim.es; 22 Hospital Universitario Central de Asturias, 33011 Oviedo, Spain; loresuarezgu@gmail.com; 23 Hospital Universitari Parc Taulí, Universitat Autònoma de Barcelona, Institut d’Investigació i Innovació Parc Taulí (I3PT), 08208 Sabadell, Spain; acaixas@gmail.com; 24 Hospital General Universitario Gregorio Marañón, Instituto de Investigación Sanitaria Gregorio Marañón (IISGM), 28007 Madrid, Spain; irenebreton@gmail.com; 25 Hospital Universitari i Politècnic La Fe, 46026 Valencia, Spain; rosacamaragomez@gmail.com; 26 Hospital Central de la Defensa Gómez Ulla, 28047 Madrid, Spain; ronapenso@hotmail.com; 27 Hospital Universitario Fundación Jiménez Díaz, 28040 Madrid, Spain; mariajocf@gmail.com; 28 Hospital Mútua de Terrassa, 08221 Terrassa, Spain; andreusimoservat@gmail.com; 29 Hospital de Basurto, 48013 Bilbao, Spain; fgonigoicoechea@gmail.com (F.G.); cristinarri@hotmail.com (C.A.); 30 Hospital Universitario Lucus Augusti, 27003 Lugo, Spain; manuelbotanal@gmail.com; 31 Hospital Universitario Reina Sofia, 14004 Córdoba, Spain; contentine@yahoo.es (A.C.); rebollo.ang@gmail.com (Á.R.)

**Keywords:** bariatric surgery, time-within-remission range, type 2 diabetes, metabolic control

## Abstract

Almost one third of patients do not achieve type 2 diabetes remission after bariatric surgery or are unable to sustain this effect long term. Our objective was to delve further into the dynamic responses of diabetes after bariatric surgery and to evaluate the “time-within-remission range” as a variable of metabolic control. A descriptive cohort study was done using a computerised multicentre and multidisciplinary registry. All data were adjusted by propensity score. A total of 1186 subjects with a follow-up of 4.5 ± 2.5 years were included. Type of surgery, diabetes remission, recurrence of diabetes, “time-within-remission range” and key predictors of diabetes outcomes were assessed. All patients (70% women, 51.4 ± 9.2 years old, body mass index (BMI) 46.3 ± 6.9 kg/m^2^) underwent primary bariatric procedures. “Time-within-remission range” were 83.3% (33.3–91.6) after gastric bypass, 68.7% (7.1–87.5) after sleeve gastrectomy and 90% (83.3–92.8) after malabsorptive techniques (*p* < 0.001 for all). Duration of diabetes, baseline HbA1c and insulin treatment were significantly negatively correlated with the “time-within-remission range”. The association of bariatric techniques with “time-within-remission range”, using gastric bypass as a reference, were: odds ratio (OR) 3.70 (2.34–5.84), *p* < 0.001 for malabsorptive techniques and OR 0.55 (0.40–0.75), *p* < 0.001 for sleeve gastrectomy. Characteristics of type 2 diabetes powerfully influence the outcomes of bariatric surgery. The “time-within-remission range” unveils a superiority of gastric bypass compared to sleeve gastrectomy.

## 1. Introduction

There is no doubt that obesity has reached pandemic trends during the latter half of the century [1]. This evidence goes in parallel with another concern, the finding of higher waist circumference at increasing body mass index (BMI) levels, further accelerating the cardiometabolic health consequences of abdominal adiposity [2]. In fact, obesity increases the risk of comorbid conditions, including type 2 diabetes, cardiovascular diseases, obstructive sleep apnea, non-alcoholic fatty liver disease and cancer. Therefore, obesity impairs quality of life and life expectancy of the world’s population, becoming a major public health challenge that requires strategies at many levels, not only on prevention but also on monitoring and management. However, the discouraging long-term results achieved with dietary and behavioural interventions and the up-till-now few safe and effective drugs available for the treatment of obesity have led to a marked increase in the use of bariatric surgery (BS) in Western countries [3].

Nowadays, BS is the most effective treatment for weight loss and remission of comorbidities in subjects with severe obesity. Regarding type 2 diabetes, BS results in superior short- and long-term outcomes compared to the best medical treatment. These outcomes include diabetes remission, and improvement in microvascular complications, together with favourable consequences in hard endpoints such as macrovascular complications and death [4,5,6]. However, a growing number of patients (25%–40%) with diabetes do not achieve “biochemical remission” after BS or are unable to sustain this effect in the long term despite initial success [7,8,9]. High baseline HbA1c, low C-peptide values, preoperative use of insulin, long diabetes duration and low magnitude of weight loss have been negatively related to type 2 diabetes remission [10].

There is still no conclusive evidence about which is the best type of surgery to treat obesity and diabetes. Three randomised clinical trials (RCT) have been carried out to assess diabetes’ short-term outcomes comparing gastric bypass (GBP) and sleeve gastrectomy (SG), with similar results [11,12,13]. In addition, the RCT from Mingrone et al. clearly showed better glycaemic control and weight loss results after biliopancreatic diversion compared to GBP and medical treatment [14]. However, the limited number of participants assigned to each arm precluded definitive conclusions. Also, there is no data available from RCT about the impact of BS on other metrics of glycaemic control beyond glycaemia and HbA1c. Data from the continuous glucose monitoring (CGM) studies in patients with type 1 diabetes suggest that time within glucose target range (“time-in-range”) provides more suitable information than fasting plasma glucose or HbA1c for medical care [15]. This kind of metric approximation has not been previously applied to BS studies. 

In May 2011, the Obesity Group of the Spanish Endocrinology and Nutrition Society (GOSEEN) created a non-exhaustive computerised multicentre and multidisciplinary registry of patients with obesity undergoing bariatric procedures in Spanish public hospitals since 2000 [16]. The RICIBA (Registro Informatizado de Cirugía Bariátrica) tries to better understand the baseline characteristics and surgical outcomes of BS [17]. Therefore, our objective was to delve further into the dynamic responses of diabetes in 1186 patients with morbid obesity and type 2 diabetes included in the RICIBA-DM (diabetes mellitus) study who underwent bariatric procedures between 2000 and 2016 in Spain. 

## 2. Methods

### 2.1. Study Design and Description of the Study Population

In this study, we have investigated the dynamic responses of diabetes following bariatric surgery in a large cohort of Spanish subjects according the “Strengthening the Reporting of Observational Studies in Epidemiology” guidelines for reporting cohort studies [18]. The RICIBA-DM was created in August 2017 with the main objective to register patients with type 2 diabetes, who underwent primary BS in 25 public Spanish hospitals between the years 2000 and 2016. The initial database was designed by a specific board from GOSEEN, and every health professional willing to participate obtained an access password. Each researcher had access only to their own patients according to the Spanish data protection law. 

Only subjects with complete and documented baseline data and at least a 1-year follow-up period were included in the final analysis. Subjects who underwent revisional surgeries finished the follow-up at that moment. 

### 2.2. Assessed Variables in the RICIBA-DM 

The following information was recorded at baseline and every six months until the end of the follow-up: demographic and anthropometric data (gender, age, weight and height), analytical data (triglycerides, high-density lipoprotein cholesterol (HDLc) and low-density lipoprotein cholesterol (LDLc)), morbidity related to the obesity state (high blood pressure, dyslipidaemia), data related with diabetes (fasting plasma glucose, HbA1c, antidiabetic treatment, need for insulin, disease duration) and type of surgical procedure performed. 

### 2.3. Study Size for the RICIBA-DM

By January 2019, a total of 1637 registers had been summited to the RICIBA-DM database. The database including the required clinical, analytical and anthropometric information of 1337 patients with obesity and type 2 diabetes was closed in February 2019. Finally, the 1186 subjects with no missing data and who had a minimum follow-up of 12 months after BS were included in the analysis (Figure S1).

### 2.4. Diagnostic Criteria to Define Metabolic and Weight Responses After Bariatric Surgery

Diabetes was diagnosed according to the American Diabetes Association criteria [19]. We defined diabetes remission as HbA1c < 6.5% and fasting plasma glucose (FPG) < 126 mg/dl in the absence of diabetic medication, including both complete and partial remission within this description. Recurrence of diabetes was defined as a new diagnosis of diabetes once remission had been achieved. Good metabolic control was defined as HbA1c < 7%, irrespective of diabetes medication [19]. A novel variable, “time-within-remission range”, was created: the time that subjects were in diabetes remission during the follow-up after BS, expressed as median percentage (25th–75th percentiles). The aim of this variable was to include all the periods of time during follow-up during which subjects met the diabetes remission criteria, taking into account that subjects could switch from one glycaemic category to another several times during the follow-up.

Hypertension was defined as blood pressure ≥ 140/90 mmHg or taking antihypertensive treatment. Dyslipidaemia was based on the presence of fasting LDLc ≥ 160 mg/dL, HDLc ≤ 40 mg/dL, fasting triglyceride ≥ 200 mg/dL or active use of lipid lowering therapy [19]. Weight regain was calculated as the difference between the weight lost at nadir and the weight on the last observation expressed as percentage [20].

### 2.5. Statistical Analysis 

Given the diversity of surgical techniques, in particular, malabsorptive surgeries, we grouped surgeries into three main groups: GBP, SG and the malabsorptive techniques’ group (MAs), which includes biliopancreatic diversion with or without duodenal switch, and single anastomosis duodenal-ileal bypass with sleeve gastrectomy (SADI-S).

Categorical variables were described as frequencies and percentages and continuous variables as mean (standard deviation, SD). The survival function was described using the Kaplan–Meier function.

Standardised differences, defined as differences between groups divided by pooled standard deviation, were used to assess heterogeneity between the three cohorts for baseline covariables. To address potential sources of bias, the Inverse Probability of the Treatment Weights (IPTW) approach [21] was used to create a pseudo-population, in which the 3 surgery groups were balanced across baseline covariates. The stabilised weights were calculated using propensity scores (PS) obtained from a logistic regression model aimed at minimising the standardised differences between arms [22]. The covariates included in the final model were age, gender, BMI, weight, glucose, LDLc, HDLc, triglycerides, HbA1c, type of diabetes treatment, diabetes duration, hypertension and dyslipidaemia. Almost all are key predictors of diabetes outcomes that might be imbalanced in non-randomised comparisons.

Post-baseline variables and outcomes were available only after the definition of the final model for the IPTW approach. Covariate balance was assessed using the standardised differences with the initial goal to achieve values <0.10. The IPTW approach was used to define insignificant differences in potential confounders. For baseline comparisons between GBP and SG, this cut-off target was always achieved. For comparisons between GBP or SG and MAs, since it was unfeasible to achieve <0.1 for all variables and that for some authors, <0.2 might also be acceptable, the cut-off value for this comparison was redefined to 0.15 [23].

Baseline categorical data were compared using the chi-square test and continuous variables using analysis of variance (ANOVA) with rank-transformed data for raw and IPTW-adjusted analyses. Raw and IPTW-adjusted logistic and Cox regression models were used to estimate risks: odds ratio (OR) and hazard ratio (HR) with 95% confidence interval (CI) for binary and time to event variables, respectively. 

Since a number of variables were different at baseline among the types of surgery performed, all data shown in this paper were adjusted by propensity score using the IPTW method, unless specified otherwise. In all statistical analyses, a two-sided type-I error of 5% was applied. The software SPSS v25 (IBM) and SAS v9.4 (Cary, NC, USA) were used. 

### 2.6. Ethics Statement 

A non-written informed consent was defined in the RICIBA-DM protocol and was obtained from all participants. The human ethics committee of Hospital Clínic de Barcelona approved the study and the procedure outlined for the verbal consent obtainment. The entire process was documented in the clinical history of each participant. Each researcher had access only to their own patients, as the system prevented unauthorised access by third parties, according to the Spanish data protection law. Finally, all patient records and information were anonymised and de-identified prior to analysis. 

## 3. Results

### 3.1. Baseline Characteristics of the Whole Cohort and According to Bariatric Surgery

A total of 1186 individuals with type 2 diabetes who underwent BS between the years 2000–2016 in 25 public Spanish hospitals were included. The average age was 51.4 ± 9.2 years, presurgical BMI was 46.3 ± 6.9 kg/m^2^ and 70.0% were women. The known duration of diabetes was 6.3 ± 5.7 years and baseline mean HbA1c was 7.4% ± 1.8%. Two-hundred and nineteen individuals (18.5%) were treated only with diet, 604 (50%) with non-insulin medications, 229 (19.3%) with both insulin and non-insulin medications and 134 (11.3%) exclusively with insulin. 

Of all patients, 47.5% underwent GBP, 35.8% SG and 16.5%, MAs. The length of follow-up for the whole cohort was 4.5 ± 2.5 years (4.4 ± 2.4 in GBP, 4.0 ± 2.0 in SG and 4.1 ± 3.0 in MAs). The rate of follow-up was 100% at 1 year, 93.6% at 2 years and 49% after 5 years of BS, without differences between surgical techniques. The main baseline clinical and biochemical characteristics, including comorbidities, for the whole cohort and according to BS technique, before and after propensity score-IPTW approach, are shown in Table S1 and Table 1, respectively.

### 3.2. Diabetes Remission 

Diabetes remission rates in the whole cohort were: 72% (95%CI: 69–74) at 1 year of surgery, 76% (74–79) at 2 years and 80.3% (78–82) at 5 years. Remission rates by type of surgery were as follows: after GBP, the remission rate was 73% (69–76) at 1 year, 79% (75–82) at 2 years and 83% (79–86) at 5 years, after SG, 64% (60–69), 68% (64–73) and 74% (69–78) and after MAs, 84% (78–89), 87% (82–91) and 89% (84–93) at 1, 2 and 5 years, respectively. 

Multivariate analysis for diabetes remission at one year after BS shows that remission was associated with: age at the time of BS (OR 0.97, (0.96–0.99), *p* = 0.006), diabetes duration (OR 0.89, (0.86–0.92), *p* < 0.0001), presurgical BMI (OR 0.97, (0.94–0.99), *p* = 0.004), baseline HbA1c (OR 0.72, (0.65–0.79), *p* < 0.0001), insulin treatment (OR 0.23, (0.12–0.42), *p* < 0.0001) and weight loss 1 year after BS (OR 1.05, (1.03–1.07), *p* < 0.0001). Regarding the type of BS, using GBP as the reference, associations with remission rates were as follows: (OR 3.69 (2.14–6.35), *p* < 0.0001) for MAs and (OR 0.73 (0.52–1.02), *p* = 0.068) for SG. 

**Figure 1:**
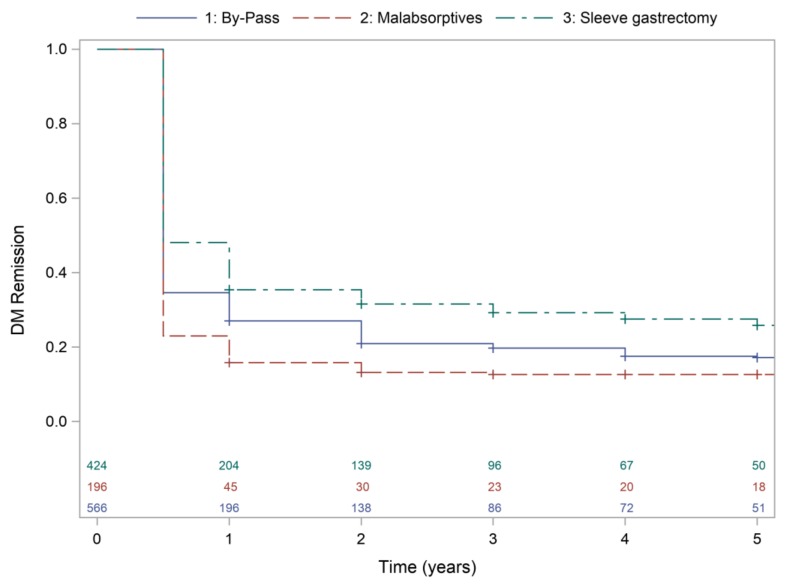
Kaplan–Meier for type 2 diabetes remission according to type of bariatric surgery.

Multivariate IPTW Cox survival analysis (Concordance statistic (95%CI): 0.80 (0.78–0.82)) shows that type of surgery, diabetes duration, baseline insulin treatment, baseline HbA1c and percentage of weight loss at 1 year were independent factors associated to diabetes remission along the follow-up. HR were 0.91 (0.78–1.05) for SG and 1.19 (1.00–1.42) for MAs (GBP as reference), 0.96 (0.95–0.97) for diabetes duration, 0.58 (0.43–0.79) for insulin treatment, 0.92 (0.90–0.96) for HbA1c and 1.01 (1.02–1.20) for weight loss at 1 year. Figure 1 shows survival curves for diabetes remission by type of surgery.

### 3.3. Time-in-Remission Range

“Time-within-remission range” expressing the percentages of persistence of diabetes remission during all the follow-up periods, were 83.3% (33.3–91.6) after GBP, 68.7% (7.1–87.5) after SG and 90% (83.3–92.8) after MAs (*p* < 0.001 for comparisons between the three groups). 

Previous duration of diabetes was negatively correlated with the percentage of “time-within-remission range”, OR 0.89, (0.86–0.92), *p* < 0.0001, as was baseline HbA1c, OR 0.85, (0.74–0.97), *p* = 0.0184 and treatment with insulin, OR 0.24, (0.17–0.35), *p* < 0.0001. However, age and baseline BMI were not related with this parameter. Weight lost at 1 year after BS was positively correlated, OR 1.06, (1.04–1.08), *p* < 0.0001, with the “time-within-remission range”. The association of BS type with “time-within-remission range”, using GBP as a reference, were: OR 3.70 (2.34–5.84), *p* < 0.0001 for MAs and OR 0.55 (0.40–0.75), *p* = 0.0002 for SG.

### 3.4. Good Metabolic Control

Good metabolic control was presented in 92%, 89% and 86% patients in the whole cohort at 1, 2 and 5 years of follow-up after BS, respectively. Good metabolic control at 1 year was negatively associated with age, OR 0.96 (0.93–0.99), *p* = 0.003, diabetes duration, OR 0.93, (0.90–0.97), *p* < 0.0001, baseline treatment with insulin, OR 0.12 (0.03–0.43), *p* = 0.001 and baseline HbA1c, OR 0.71 (0.63–0.80), *p* < 0.001. Regarding the type of surgery, compared to GBP, the associations were: OR 4.91 (1.67–14.45), *p* = 0.004 for MAs, and OR 0.53 (0.32–0.86), *p* = 0.011 for SG. 

### 3.5. Diabetes Recurrence 

After diabetes remission, recurrence occurred in 16% (12–19, 95%CI) of patients after GBP, 24% (18–28, 95%CI) after SG and 12% (12–19, 95%CI) after MAs. Recurrence was associated with diabetes duration (hazard ratio (HR) 1.04, (1.02–1.07), *p* = 0.001), baseline HbA1c (HR 1.14, (1.11–1.25), *p* = 0.002), insulin treatment (HR 2.35, (1.38–4.0), *p* < 0.0001), weight regain (HR 1.01, (1.01–1.02), *p* = 0.03) and type of BS (HR 0.48 (0.29–0.82), *p* = 0.007 for MAs and HR 1.56 (1.13–2.16), *p* = 0.006 for SG, using GBP as reference).

### 3.6. Weight Loss and Weight Regain 

**Figure 2:**
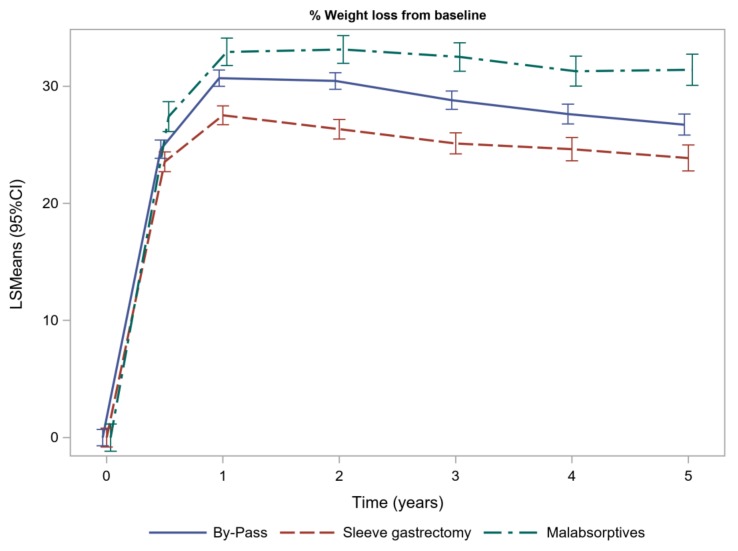
Weight loss evolution of patients with type 2 diabetes, expressed as percentage of initial body weight.

One year after BS, the estimated weight loss was 30.7% (29.9–31.4) after GBP, 27.52% (26.72–28.32) after SG and 32.93% (31.76–34.10) after MAs. After 1 year of follow-up, weight loss was statistically different between the three types of surgery included in our cohort. Figure 2 shows the weight loss evolution. 

Weight regain at three years was 14.78% (13.2–16.37) after GBP, 10.87% (8.41–13.3) after MAs and 18.99% (17.14–20.84) after SG. Weight regain was statistically different between the three types of BS. A 20% weight regain at three years after BS was observed in 18.9% after GBP, 26.7% after SG and 13.8% after MAs. All comparisons between type of BS were statistically different, *p* < 0.05. Table 2 shows the data regarding weight loss and weight regain. 

## 4. Discussion

This study involved a large cohort of post-bariatric patients with type 2 diabetes from public hospitals in a Mediterranean area. After matching using the PS and the IPTW methods, we examined the diabetes and weight loss outcomes of the three main types of surgical techniques used in BS. In our cohort, MAs was the group with higher diabetes remission, a major percentage of “time-within-remission range”, less diabetes recurrence, more weight loss and less weight regain compared to GBP. On the other side, subjects who had undergone SG obtained similar diabetes remission rates but spent less “time-within-remission range”, achieved less percentage of good metabolic control and had more recurrences, together with a smaller weight loss and more weight regain compared to GBP.

This study has several strengths. First, the use of data obtained from clinical registries, albeit in a retrospective way, may better reflect the daily clinical practice compared to results from randomised control trials, which permits the generalisation of the results to our population. Another strong point is the excellent adjustment for covariates, performed taking into consideration several known variables relevant to the prediction of diabetes outcomes, thus allowing the different types of surgeries to be compared without the interference of confounding factors. Also, it is extremely interesting that we have included a considerable number of patients operated on using malabsorptive techniques. Finally, we introduced a new approach to measure the metabolic beneficial effects of BS, the percentage of time during the follow-up period that diabetes remains under remission. Taking into account that the legacy effect also occurs in type 2 diabetes [24], our study attempts to transpose the glucose parameter “percentage of time-in-range” used in CGM in type 1 diabetes to BS results. Given that, this variable considers all the time during which the subject remains in each glycaemic category and not just the time until the first diabetes recurrence, while encompassing the different patterns of weight loss and weight recovery. The “time-within-remission range” emerges as a relevant parameter to evaluate the long-term diabetes outcomes, a chronic disease largely conditioned by the fluctuations in weight that occur throughout the life of the subjects after BS. However, we acknowledge that the potential implications for daily clinical practice still need further evaluation.

Two population-based cohorts that were matched with no surgical management show results from real world settings in Europe, using stricter diabetes remission criteria [25,26]. A Danish cohort, with 1111 patients with diabetes, found a one-year diabetes remission rate of 74% and a relapse rate of 27% after 5 years of follow-up. However, GBP was the only surgical technique included [25]. In another cohort from the United Kingdom, Yska et al. found greater remission of diabetes after GBP (*n* = 280) than after SG (*n* = 83) or gastric band (*n* = 200) [26]. Neither of the two studies analysed other relevant issues, such as the duration of diabetes remission, the recurrence or the percentage of good metabolic control. A third real world setting study, focussing only on medication discontinuation in the short term, showed that GBP (*n* = 922) was more effective than SG (*n* = 1111) in achieving this primary outcome [27]. None of these studies evaluated the factors associated with diabetes outcomes exhaustively. On the other hand, our cohort, like others, showed that MAs has the greatest efficacy regarding weight loss and diabetes remission. But, given the concern about nutritional consequences, it requires a very personalised indication [28,29].

In our Spanish cohort, characteristics related with type 2 diabetes, such as longer diabetes duration, need for insulin therapy and higher baseline HbA1c values, were strongly associated with all assessed outcomes (remission, good metabolic control, time-within-remission range and recurrence). In addition, some features of the patient such as older age and higher BMI were also negatively related with diabetes remission. Finally, weight response after BS was also able to influence type 2 diabetes remission (weight loss) and recurrence (weight regain). The same predictors were described in previous studies [30,31].

Our results regarding weight loss were comparable to a recently published US large PS-matched bariatric cohort (*n* = 8493 GBP and *n* = 4387 SG). Their results showed that individuals with diabetes lost more weight after GBP than SG, but diabetes outcomes results were not published [32]. Our data shows a greater weight regain after SG than GBP and MAs, in the same direction as an Indian cohort (*n* = 9617), although their data were not adjusted for baseline differences and data on the percentage of diabetes was not provided [33]. On the other hand, a Spanish cohort recently reported a high percentage of reoperations after SG, expressly 23%, due to issues regarding gastroesophageal reflux or insufficient weight loss; however, this may be an advantage of GBP over SG [31].

Our study has some limitations. First, patients were not randomly assigned to the procedures, so there was a risk of unobserved confounding that may have persisted despite covariate and PS adjustment in our pair-wise comparisons. Second, the short duration of follow-up and attrition could preclude recognition of diabetes relapse that could hinder long-term generalisation. Lastly, the adverse effects occurring with the three types of surgery included in the study have not been assessed, precluding an accurate assessment of cost effectiveness ratio.

## 5. Conclusions

In conclusion, our results from a large multicentre Spanish cohort showed that diabetes outcomes and weight evolution were better after MAs surgeries compared to GBP and SG. Although SG showed remission rates like GBP, patients undergoing SG experienced a greater recurrence of diabetes, a shorter time-within-remission range and a lower percentage of good metabolic control. Furthermore, SG was also associated with less weight loss and greater weight recovery than GBP. Based on our findings, we propose choosing the bariatric technique that achieves the best metabolic effect, the greatest weight loss and the longest time of diabetes remission, carefully weighed with the adverse effects and the inherent surgical risk. For this selection, it is imperative to assess the characteristics of type 2 diabetes, as they will powerfully influence the outcomes of BS.

## Figures and Tables

**Table 1:** Baseline main clinical and metabolic characteristics of participants in the study after propensity score adjustment.

	Treatment			*p*-Value			Standardised Difference		
	GBP (*n* = 562)	SG (*n* = 423)	MAs (*n* = 196)	GBP Versus SG	GBP Versus MAs	SG Versus MAs	GBP Versus SG	GBP Versus MAs	SG Versus MAs
Gender (%females)	69.2	70.0	74.4	0.797	0.173	0.262	−0.017	−0.114	−0.098
Age (years)	51.3 (8.4)	51.3 (9.9)	52.1 (10.2)	0.909	0.176	0.209	−0.005	0.089	0.087
Weight (Kg)	121(20)	121(22)	121 (22)	0.633	0.944	0.784	−0.008	0.006	0.014
BMI (Kg/m^2^)	46.1 (6.7)	46.0 (6.9)	46.4 (6.5)	0.949	0.453	0.508	−0.011	0.043	0.053
Dyslipidaemia (%)	48.3	49.2	48.6	0.771	0.944	0.881	0.019	0.006	−0.013
Hypertension (%)	70.4	72.5	76.8	0.481	0.089	0.261	0.045	0.144	0.098
Diabetes duration (years)	6.2 (5.5)	6.1 (5.6)	6.3 (6.0)	0.625	0.846	0.781	−0.021	0.016	0.036
On Insulin (%)	11.3	10.5	8.5	0.981	0.714	0.843	0.027	0.104	0.108
Good control (%)	50.9	51.6	55.4	0.838	0.283	0.380	0.013	0.089	0.076
HbA1c (%)	7.3 (1.7)	7.3 (1.8)	7.3 (1.6)	0.891	0.906	0.977	0.007	−0.026	−0.032
HbA1c (mmol/mol)	56 (18.6)	56 (19.7)	56 (17.5)	0.891	0.906	0.977	0.007	−0.026	−0.032
FPG (mg/dL)	159 (59)	159 (65)	157 (53)	0.388	0.857	0.387	−0.001	−0.033	−0.030
Triglycerides (mg/dl)	183 (128)	183 (128)	175 (138)	0.974	0.343	0.274	0.003	−0.056	−0.059
LDLc (mg/dl)	106 (34)	106 (36)	105 (35)	0.822	0.917	0.938	−0.008	−0.031	−0.023
HDLc (mg/dl)	45 (14)	45 (13)	46 (11)	0.545	0.340	0.148	−0.005	0.057	0.065

Data are expressed as median (standard deviation) or percentage. BMI: body mass index; FPG: fasting plasma glucose; HbA1c: glycated haemoglobin; LDLc: low-density lipoprotein cholesterol; HDLc: high-density lipoprotein cholesterol; GBP: gastric bypass; SG: sleeve gastrectomy; MAs: malabsorptive surgeries.

**Table 2:** Weight loss and weight regain evolution bariatric surgery according to the surgical technique, Inverse Probability of the Treatment Weights (IPTW) analysis.

	Time After BS	Treatment			*p*-Value		
		GBP	SG	MAs	GBP Versus SG	GBP Versus MAs	SG Versus MAs
Weight loss * (%)	0.5 year	24.6 (23.8–25.4)	23.5 (22.6–24.4)	27.4 (26.1–28.7)	0.064	0.003	<0.001
	1 year	30.6 (30.0–31.4)	27.5 (26.7–28.3)	32.9 (31.7–34.1)	<0.001	0.012	<0.001
	2 years	30.4 (29.7–31.2)	26.3 (25.5–27.1)	33.1 (31.9–34.3)	<0.001	0.001	<0.001
	3 years	28.8 (28.0–29.6)	25.2 (24.2–26.0)	32.5 (31.3–33.7)	<0.001	<0.001	<0.001
	4 years	27.6 (26.7–28.5)	24.6 (23.6–25.6)	31.2 (30.0–32.7)	<0.001	<0.001	<0.001
	5 years	26.7 (25.8–27.6)	23.8 (22.7–25.0)	31.4 (30.1–32.7)	<0.001	<0.001	<0.001
Weight regain ^&^ (%)	2 years	6.5 (5.0–7.9)	10.8 (9.1–12.6)	5.9 (3.5–8.5)	0.002	0.734	0.02
	3 years	14.7 (13.2–16.4)	18.9 (17.1–20.8)	10.8 (8.4–13.3)	0.007	0.008	<0.001
	4 years	19.7 (17.9–21.4)	24.7 (22.7–26.8)	14.7 (12.1–17.2)	0.002	0.001	<0.001
	5 years	22.4 (20.6–24.2)	28.7 (26.6–31.0)	15.2 (12.7–17.8)	<0.001	<0.001	<0.001

Data are expressed as mean (95%CI). * Expressed as percentage of initial body weight. ^&^ Expressed as percentage of weight lost at nadir point. BS: bariatric surgery; GBP: gastric bypass; SG: sleeve gastrectomy; MAs: malabsorptive surgeries.

## Supplementary Materials

The following are available online at https://www.mdpi.com/2077-0383/9/4/1070/s1: Table S1: Baseline main clinical and metabolic characteristics of participants in the study before propensity score adjustment. Figure S1: Flow chart of the study population.
