# Supplementary material for: New Metrics to Assess Type 2 Diabetes after Bariatric Surgery: The “Time-Within-Remission Range”
Source: J Clin Med. 2020 Apr 9;9(4):1070. doi: 10.3390/jcm9041070 (PMC7230819; doi:10.3390/jcm9041070)
Supplement: Supplementary file 1 [file jcm-09-01070-s001.pdf]

Table S1. Baseline main clinical and metabolic characteristics of participants in the study before propensity score adjustment.

	Total Cohort (n=1186)	Treatment			p value			Standardized Difference		
		GBP (n=566)	SG (n=424)	MAs (n=196)	GBP vs. SG	GBP vs. MAs	SG vs. MAs	GBP vs. SG	GBP vs. MAs	GBP vs. SG
**Gender (%females)**	69.9	72.8	68.6	64.3	0.154	0.024	0.284	0.092	0.184	0.092
**Age (yrs)**	51.4 (9.2)	50.8 (8.6)	52.9 (9.4)	50.0 (9.8)	0.001	0.384	0.001	0.228	-0.091	-0.301
**Weight (Kg)**	121 (22)	118 (19)	122 (23)	127 (23)	0.016	<0.001	0.007	0.185	0.405	0.204
**BMI (Kg/m²)**	46.2 (6.9)	45.5 (6.4)	46.3 (7.1)	48.4 (7.5)	0.099	<0.001	0.002	0.112	0.411	0.288
**Dyslipidaemia (%)**	48.2	38.3	55.7	60.7	<0.001	<0.001	0.237	0.352	0.459	0.103
**Hypertension (%)**	70.2	64.5	74.8	76.5	0.001	0.002	0.635	0.225	0.266	0.041
**Diabetes duration (yrs)**	6.2 (5.7)	5.9 (5.2)	6.8 (6.1)	5.9 (6.4)	0.046	0.116	0.006	0.154	-0.008	-0.147
**On Insulin (%)**	11.3	13.8	7.3	12.8	<0.001	0.917	<0.001	0.464	0.056	0.445
**Good metabolic control (%)**	50.5	52.3	50.9	44.4	0.673	0.056	0.129	-0.027	-0.159	-0.132
**HbA1c (%)**	7.4 (1.8)	7.3 (1.8)	7.4 (1.8)	7.6 (1.8)	0.464	0.009	0.048	0.035	0.187	0.153
**HbA1c (mmol/mol)**	57 (19.7)	56 (19.7)	57 (19.7)	60 (19.7)	0.464	0.009	0.048	0.035	0.187	0.153
**FPG (mg/dL)**	160 (61)	162 (61)	154 (61)	164 (60)	0.005	0.641	0.012	-0.133	0.022	0.157
**Triglycerides (mg/dl)**	183 (129)	184 (130)	181 (121)	187 (145)	0.976	0.697	0.685	-0.024	0.019	0.042
**LDLc (mg/dl)**	107 (35)	109 (34)	106 (37)	103 (35)	0.179	0.073	0.490	-0.062	-0.149	-0.082
**HDLc (mg/dl)**	45 (13)	46 (13)	45 (13)	45 (11)	0.017	0.528	0.217	-0.096	-0.052	0.052

Data are expressed in percentages (%) or mean (standard deviation). BMI: body mass index; FPG: fasting plasma glucose; GBP: gastric bypass; SG: sleeve gastrectomy; MAs: malabsorptive surgeries.

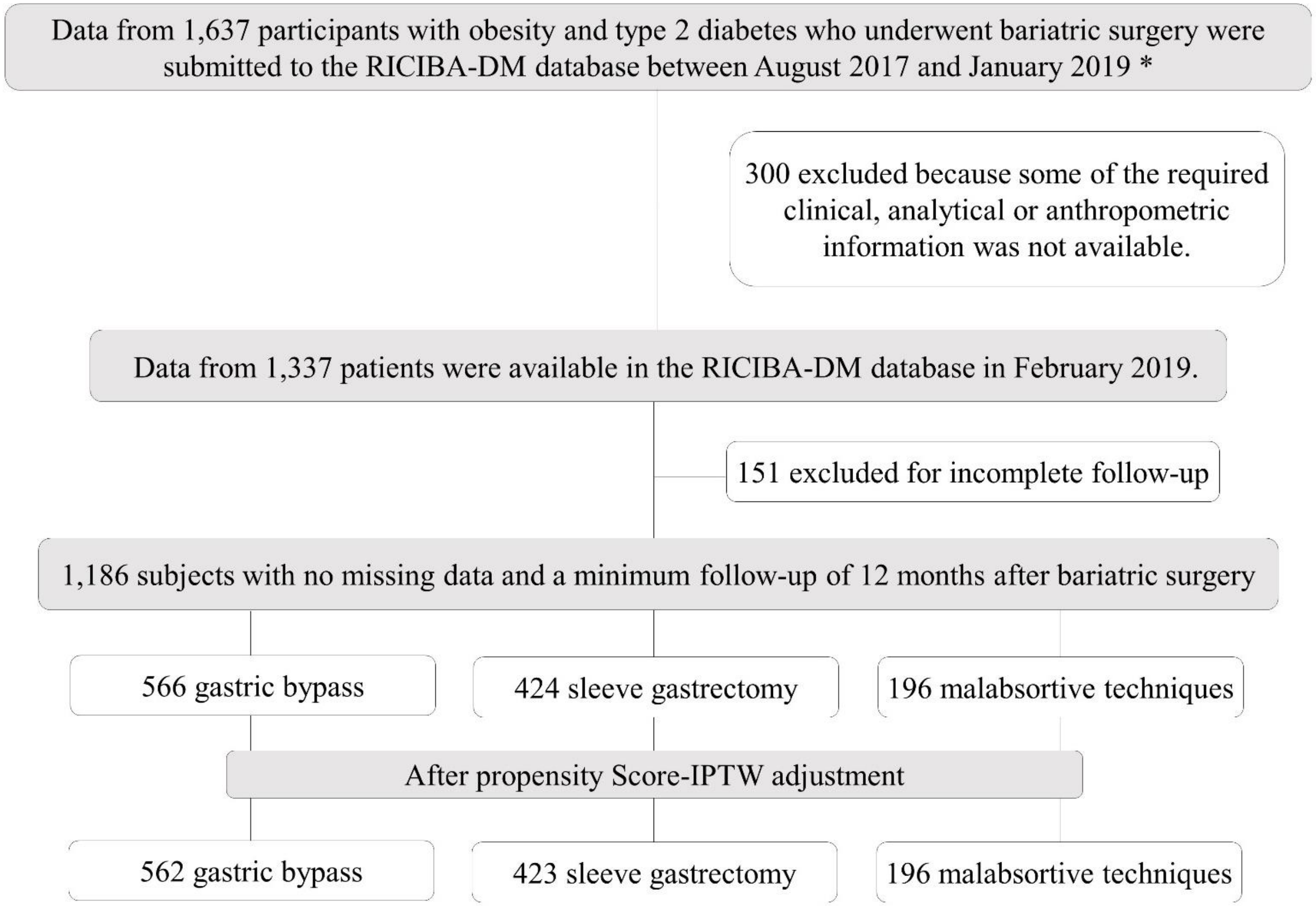

Figure S1. Flow chart of the study population. * There is no data regarding the number of patients who declined to participate in the study; IPTW: Inverse Probability of the Treatment Weights (IPTW).
